# Isolation of Lytic Bacteriophages of *Escherichia coli* and Their Combined Use with Antibiotics Against the Causative Agents of Colibacillosis in Calves

**DOI:** 10.3390/vetsci12090817

**Published:** 2025-08-26

**Authors:** Pavel G. Alexyuk, Andrey P. Bogoyavlenskiy, Kuralay S. Akanova, Yergali S. Moldakhanov, Timur T. Kerimov, Nadezhda S. Sokolova, Vladimir E. Berezin, Madina S. Alexyuk

**Affiliations:** Research and Production Center for Microbiology and Virology, Almaty 050010, Kazakhstan; alpagen@live.com (P.G.A.); anpav_63@mail.ru (A.P.B.); vberezin359@gmail.com (V.E.B.)

**Keywords:** colibacillosis, bacteriophage, *Escherichia coli*, antibiotic resistance, phage therapy, synergy, whole-genome sequencing

## Abstract

The spread of antibiotic-resistant pathogenic *Escherichia coli* poses a serious threat to calf health on livestock farms. With the decline in antibiotic therapy effectiveness, alternative approaches such as phage therapy are urgently needed. This study aimed to isolate lytic *E. coli* bacteriophages, characterize their properties, and evaluate the synergistic effects of their combined use with veterinary antibiotics against colibacillosis pathogens in calves. As a result of the work, 4 bacteriophages were isolated from wastewater from various cities of Kazakhstan: vB_EcoS_ABO/4, vB_EcoM_PL/4, vB_Eco_CWW/26, vB_EcoM_ShWW/46. Morphological, biological, and genomic analyses showed that the phages belong to different genera of the *Caudoviricetes* class, possess high lytic activity, broad host range, environmental stability, and lack genes associated with lysogeny, antibiotic resistance, or virulence. Interaction studies with antibiotics revealed synergistic or additive effects in over 75% of cases. These findings highlight the strong potential of the isolated bacteriophages for independent or adjunctive use in the treatment and prevention of colibacillosis in calves. However, further in vivo studies are required to definitively confirm their therapeutic efficacy.

## Simple Summary

Colibacillosis is a serious infectious disease caused by *Escherichia coli*, which commonly affects calves and can result in significant economic losses in livestock production. The reduced effectiveness of antibiotics due to increasing bacterial resistance necessitates the development of alternative therapeutic approaches. In this study, we isolated four bacteriophages—viruses that infect and lyse bacteria—from wastewater samples collected in various cities across Kazakhstan. These phages demonstrated strong lytic activity against pathogenic bacterial strains, rapid replication, environmental stability, and high efficacy in bacterial destruction. Furthermore, the combined application of bacteriophages and conventional antibiotics resulted in a synergistic effect, leading to more rapid suppression of bacterial growth in most cases. Whole-genome sequencing revealed that the isolated phages lacking any genes associated with bacterial virulence or antibiotic resistance, indicating their biosafety for potential therapeutic use. Taken together, our findings support the possible potential of newly isolated bacteriophages as effective and safe agents for the control of bacterial infections in animals, and suggest their utility in reducing antibiotic dependency in livestock production.

## 1. Introduction

Livestock farming is a strategic sector of the agro–industrial complex, with significant potential for sustainable agricultural development [1]. It not only serves as a food source, meeting the needs of almost a billion people, but is also an important element of global progress in the agricultural sector. In recent decades, the global food system has demonstrated a steady increase in the consumption of animal products [2]. In developing countries, the average annual growth rates of the meat and dairy sectors since 1970 have been 5.1% and 3.6%, respectively.

In the Republic of Kazakhstan, livestock farming occupies a key position in the structure of the agricultural economy, being a traditional and priority sector. Approximately 180 million hectares of pasture lands form unique prerequisites for the intensification of this sector. The share of livestock products in the gross volume of agricultural production is about 47%, which emphasizes their economic and social significance as a source of food security and export potential. Particular importance is attached to the breeding of cattle, which provides the country with meat and milk, and is the main export-oriented segment [3,4,5].

One of the main factors hindering the development of animal husbandry is the high incidence of gastrointestinal infections of bacterial etiology in young animals. The most common causative agents include pathogenic strains of *E. coli*, *Salmonella* spp., *Shigella* spp., among others. Of particular epidemiological importance is colibacillosis, caused by pathogenic serotypes of *E. coli*. The disease primarily affects calves between 1 and 10 days of age and is characterized by severe diarrhea, dehydration, septicemia, and, in some cases, involvement of the central nervous system [6,7].

Antibiotic therapy remains the traditional method of treating intestinal infections in cattle. Antimicrobial preparations are used both for therapeutic and prophylactic purposes, and as growth promoters. However, their irrational and uncontrolled use has led to the formation and spread of strains with multiple drug resistance, including to priority and critically important antibiotics: fluoroquinolones, macrolides, cephalosporins of the third and fourth generations, glycopeptides and polymyxins. In addition to the direct therapeutic effect, antibiotics exert selective pressure on the intestinal microflora, including opportunistic microorganisms such as *E. coli*, contributing to the spread of antibiotic resistance [8,9]. Antimicrobial resistance emerging in livestock production poses a serious threat not only to animal health but also to human health, within the framework of the global interdisciplinary One Health concept, which views the health of humans, animals, and the environment as interconnected components of a single system [10]. Transfer of resistant bacteria and resistance genes from animals to humans can occur through food, water, soil, direct contact, or through circulation in the environment [11]. This increases the risk of human infections that are difficult to treat with conventional antibiotics, making antibiotic resistance a widespread issue that impacts multiple sectors, including human healthcare, veterinary medicine, agriculture, and environmental protection. Thus, control of the use of antimicrobial drugs in veterinary medicine and the development of alternative treatment strategies, including phage therapy, are becoming key measures in the fight against the spread of resistant pathogens.

In addition, the problem of antibiotic resistance is further aggravated by the extremely slow pace of research, development, and introduction of new antibiotics. In this regard, interest has significantly increased in alternative means of combating bacterial infections, which include bacteriophages—viruses that can selectively infect and lyse host bacteria. According to a review prepared by the UK Department of Health and the Wellcome Trust, three out of the ten most promising alternatives to antibiotics are associated with the use of phages or their components [12,13]. A growing body of research has consistently demonstrated and validated the therapeutic potential of bacteriophages in the treatment of bacterial infections [14,15,16,17,18,19,20].

Phage therapy has several significant advantages over antibiotics: inexhaustibility of the resource—phages are presented in a huge variety in nature, which facilitates their search and selection for a specific pathogen; safety—lytic bacteriophages do not have a toxic effect on eukaryotic cells and are safe for the animal organism; self-limitation—phages remain in the body until the pathogen is completely eradicated, after which they are naturally excreted; the ability to self-reproduce—during the lysis process, phages exponentially increase their concentration directly at the site of infection; high specificity—the effect of phages is directed exclusively at pathogenic bacteria, while the commensal microflora remains intact; and effectiveness against antibiotic-resistant strains—phages retain lytic activity against with MDR bacteria [21,22,23,24,25].

The development of phage-based preparations is particularly relevant for Kazakhstan, where the problem of antibiotic resistance is intensified by the limited scope of research in the field of alternative antimicrobial strategies. The richness of the republic’s natural and microbiological resources offers significant potential for the isolation and characterization of lytic phages. A comparative analysis of the effectiveness of traditional antibiotic therapy and phage therapy has shown that in the presence of multiple drug resistance, the latter retains bactericidal properties. In addition, phages are able to coevolve with bacterial targets, providing long-term resistance to pathogen mutations. Work on the isolation and study of new lytic bacteriophages, the formation of extensive libraries on their basis, and the combination of traditional methods of therapy with phage therapy can significantly expand the spectrum of action and increase the effectiveness of the fight against bacterial infections.

The aim of the presented studies was to isolate lytic bacteriophages of *E. coli*, study their properties, and evaluate the synergistic effect of their combined use with traditional veterinary antibiotics against the causative agents of colibacillosis in calves, which will increase the effectiveness of the prevention and treatment of bacterial infections and reduce the risk of developing antibiotic resistance.

## 2. Materials and Methods

### 2.1. Bacterial Strains

In this study, 35 *E. coli* strains were used from the collection of the Research and Production Center of Microbiology and Virology (Almaty, Kazakhstan). These strains had been previously isolated from fecal samples of calves with diarrhea (Table S1). Antibiotic susceptibility of *E. coli* strains was determined according to CLSI and CASFM standards (Table S2).

### 2.2. Isolation of Bacteriophages

Bacteriophages were isolated from wastewater samples collected at wastewater treatment plants in the cities of Almaty, Shymkent, Konayev and Talgar, Republic of Kazakhstan. Samples were obtained in sterile 500 mL containers from a depth of 15–20 cm. The collected samples were transported and stored at 4–8 °C in light-protected packaging. Purification was performed using sequential steps: centrifugation at 3000× *g* for 30 min, followed by filtration of the supernatant through a paper filter and then through a 0.45 μm pore-size membrane filter (Sartolab^®^ RF 500, Sartorius, Göttingen, Germany).

The bacteriophages from the samples were enriched by incubating them with bacterial host cultures [26]. 1 mL of 10-fold nutrient broth (NB) (Nutrient Broth, Himedia, Thane, India), 1 mL of bacterial suspension in the exponential growth stage and 100 μL of 1% MgSO_4_ were added to 10 mL of purified sample. The resulting mixture was incubated at 37 °C for 24 h, after which it was centrifuged for 30 min at 5000× *g*, the supernatant was filtered through a 0.45 μm bacterial filter (Nalgene Syringe Filter, Thermo Scientific, Waltham, MA, USA). Purified samples were stored under sterile conditions at 4–8 °C [27].

### 2.3. Determination of Bacteriophage Titer

The bacteriophage titer was determined using the Gratia method (double-layer agar technique) [28]. Phage-containing samples were titrated 10-fold, then 1 mL of the sample and 1 mL of a suspension of the bacterial test culture with a turbidity index of 0.5 McF (≈10^8^ CFU/mL) were added to 3 mL of melted 0.7% nutrient agar (NA). The resulting mixture was poured onto the surface of solidified 2% NA and incubated at 37 °C for 24 h. After incubation, the formation of plaque forming units (PFU) at the maximum dilution was visually recorded. Sterile phosphate-buffered saline (PBS) was used as a control instead of the phage-containing sample.

### 2.4. Plaque Purification and Amplification of Bacteriophages

Pure bacteriophage lines were obtained using a method of sequential selective passages with the isolation of individual plaques [29]. Upon initial detection, isolated plaques at the highest dilution were excised from the agar medium. Phages were eluted from the plaques, propagated, and re-inoculated onto double-layer agar after 10-fold titration, followed by repeated selection of plaques at the highest dilution. This procedure was repeated three times to obtain pure bacteriophage lines.

Bacteriophages were propagated by co-cultivating phage-containing samples with the host bacterial culture. 5 mL of the host bacterial suspension in the exponential growth stage and 500 μL of the phage-containing sample were added to 40 mL of sterile NB, and the resulting mixture was incubated at 37 °C for 24 h. Following incubation, the culture mixture was centrifuged (5000× *g*, 30 min), and the supernatant was filtered through sterile 0.45 μm membrane filters to remove bacterial cells. The resulting phage lysates were stored under sterile conditions at 4–8 °C.

### 2.5. Concentration of Bacteriophages by Ultracentrifugation

Bacteriophages were precipitated from purified phage lysates by centrifugation at 100,000× *g* at 4 °C for 120 min in an Optima XPN-80 centrifuge (Beckman Coulter, Brea, CA, USA). The resulting bacteriophage pellet was dissolved in a minimum volume of sterile PBS and stored under sterile conditions at 4–8 °C. [30].

### 2.6. Electron Microscopy

Concentrated bacteriophage samples were applied to copper grids (300 mesh) coated with Formvar (Polysciences, Inc., Hirschberg an der Bergstrasse, Germany), incubated for 5 min, washed with distilled water and stained with 3% phosphotungstic acid solution (pH 6.8). Microcopy was performed on a transmission electron microscope HT7800 (Hitachi, Tokyo, Japan) at a voltage of 80 kV [31].

### 2.7. Multiplicity of Infection (MOI)

MOI of isolated phages was determined by mixing a suspension of the host bacterium at a concentration of 10^8^ CFU/mL with phage-containing samples in ratios of 10^−6^, 10^−5^, 10^−4^, 10^−3^, 10^−2^, 10^−1^, 1 and 10 PFU/CFU. The obtained samples were incubated for 4 h at 37 °C, after which they were centrifuged, filtered through 0.45 μm bacterial filters and the phage titer was determined. Samples mixed with sterile PBS instead of a bacterial suspension or bacteriophage were used as a control. The PFU-to-CFU ratio that resulted in the highest phage titer was considered as the optimal multiplicity of infection [32].

### 2.8. Adsorption Assay and One-Step Growth Curve

The rate of bacteriophage adsorption was determined by mixing a suspension of the host bacterium and the corresponding bacteriophage at MOI values of 1 (CFU/PFU—10^8^/10^8^) followed by incubation at 37 °C. At incubation time points of 0, 2, 4, 6, 10 and 15 min, 100 μL were collected from the resulting mixture, each sample was centrifuged, and the titer of non-adsorbed bacteriophages was determined in the supernatant [33].

For a one-step growth assay, a mixture of the host bacterial culture and bacteriophage was prepared at an MOI of 0.1 (CFU/PFU—10^8^/10^7^) in a total volume of 6 mL. The prepared mixture was incubated for 6 min at 37 °C, then centrifuged, the resulting pellet was resuspended in 10 mL of NB and incubated for 60 min at 37 °C. At point 0 and every 10 min of incubation, 1 mL samples were taken, passed through a bacterial filter, and the bacteriophage titer was determined in the resulting filtrates. Assessment of the latent period duration and burst size was performed using the one-step growth curve method [34].

### 2.9. Thermal and pH Stability of the Isolated Bacteriophages

To determine temperature sensitivity, 1 mL of isolated bacteriophage samples with a titer of 10^8^ PFU/mL were incubated for an hour in a frozen state (0 °C), at room temperature, 40 °C, 50 °C, 60 °C, 70 °C, 80 °C. After incubation, the concentration of lytically active bacteriophages in each sample was assessed by the double-layer agar plaque assay.

To assess pH sensitivity, 100 μL of isolated bacteriophage samples with a titer of 10^9^ PFU/mL were dissolved in 900 μL of sterile PBS with pH values of 2, 4, 7, 10, 12, 14. The pH of PBS was adjusted to the required values with 1 M HCl or 1 M NaOH. The samples were incubated for 1 h at 37 °C, after which the titer of lytically active bacteriophages was determined by the double-layer agar plaque assay [35].

### 2.10. Assessment of the Lytic Activity Level of Bacteriophages

Isolated bacteriophages at a concentration of 10^8^ PFU/mL were titrated 10-fold and mixed with a host bacterial suspension containing 10^8^ CFU/mL to obtain MOI values from 10^−8^ to 1. 100 μL of the obtained samples and 150 μL of NB were added to the wells of a microplate and incubated for 7 h at 37 °C. Bacterial growth dynamics were assessed by changes in the optical density of the suspension at 600 nm. Microplates were scanned every 30 min during the entire cultivation period. Samples containing 50 μL of sterile PBS instead of bacteriophages were used as a control for bacterial culture growth. A sample containing 50 μL of sterile PBS instead of bacterial suspension was used as a negative control [36]. The level of lytic activity of bacteriophages was assessed by the difference between the growth of the bacterial culture in the control group and the experimental samples.

### 2.11. Host Range Determination

Samples of different *E. coli* strains were infected with isolated bacteriophages at MOI values of 1, placed in the wells of the plate and NB was added. The plate with samples was incubated at 37 °C for 7 h and scanned every 30 min at a wavelength of 600 nm. Samples without bacteriophages were used as a positive control, and samples without bacteria were used as a negative control. The ability of the studied phage to infect bacterial strains was assessed by comparing the bacterial growth in the experimental samples with that in the control group.

### 2.12. Extraction and Quality Assessment of Bacteriophage DNA

Bacteriophage DNA was extracted using the commercial PureLink Viral DNA/RNA Kit (Invitrogen, Waltham, MA, USA). To eliminate residual bacterial nucleic acids, the phage lysates were pretreated with RNase A (10 mg/mL) and DNase I (1 unit/μL) before isolation, according to the protocol described earlier [37]. Quantitative and qualitative assessment of the extracted DNA was performed using a NanoQuant plate on an Infinite 200 Pro multifunctional reader (Tecan, Männedorf, Switzerland) and a Qubit 3.0 fluorometer (Thermo Fisher Scientific, Waltham, MA, USA), respectively. The resulting DNA sample that passed the assessment was used for subsequent sequencing.

### 2.13. Whole-Genome Sequencing of Bacteriophages

The library was prepared from 1 ng of genomic DNA using the Nextera XT Library Preparation Kit (Illumina, San Diego, CA, USA). The library fragment size distribution was assessed on an Agilent 2100 Bioanalyzer (Agilent Technologies, Santa Clara, CA, USA) using the Agilent High Sensitivity DNA Kit. Sequencing was performed on the Illumina MiSeq platform using the MiSeq Reagent Kit v3, which provides paired-end reads with an average length of 300 base pairs.

### 2.14. Genome Assembly and Analysis

The quality of raw sequencing reads was assessed using FastQC (Galaxy version 0.74), followed by trimming of low-quality regions and adapter sequences with Trimmomatic (Galaxy version 0.38.1), to ensure the generation of high-quality input data for subsequent genome assembly.

Genomes were assembled using the SPAdes version 3.12.0 algorithm, with the following filtering parameters: k-mer size, coverage threshold, contig length, and error correction. The longest contig from the resulting assembly was selected as a representative genome sequence. The resulting contigs were visually inspected and manually corrected using the Geneious tool 2024.0.7, which allowed for a more accurate interpretation of the assembly results and eliminated potential errors in the automatic assembly.

PROKKA version 1.7 was used for prediction and functional annotation of coding sequences (CDS) [38]. Annotated regions were further manually edited based on comparative analysis with known phage sequences in databases. Identification and localization of regions encoding transfer RNAs (tRNAs) was performed using the tRNAscan-SE tool [39].

Completeness and structural integrity of the assembled genome were assessed using CheckV (v0.8.1) via the PhageScope platform, confirming genome suitability for further analysis.

Genome analysis for genes associated with lysogeny, antimicrobial resistance and virulence was performed using PhageLeads [40] and Phagenomics (www.phagenomics.net assessed 16 July 2025) in conjunction with BACPHLIP 0.9.6 [41], AMRFinderPlus 3.12.8 [42], VirulenceFinder 2.0.4 [43] and ABRicate Version 1.0.1 [44].

Phylogenetic analysis was performed using the VICTOR web platform (https://victor.dsmz.de, accessed on 22 July 2025), which implements genome-based phylogeny and classification of prokaryotic viruses [45]. Pairwise comparisons of nucleotide sequences were performed using the Genome-BLAST Distance Phylogeny (GBDP) method [46] with parameters recommended for the analysis of prokaryotic viruses.

Intergenomic distances were calculated using the FASTME algorithm with SPR postprocessing, based on D0, D4, and D6 formulas, which were used to construct balanced minimum evolution trees [47]. The reliability of branching was estimated based on the results of 100 pseudo-bootstrap replications for each formula. The constructed trees were rooted at the midpoint according to the Farris method [48] and visualized using the ggtree package [49].

Taxonomic boundaries at the species, genus, and family levels were determined using the OPTSIL program [50], applying the recommended clustering thresholds [45] and an F value of 0.5, which reflects the fraction of links required for cluster fusion [51].

The genomic maps of the studied bacteriophages were generated using Proksee software, designed for the analysis and visualization of prokaryotic genomes [52].

### 2.15. Assessment of the Synergistic Effect

Synergy was evaluated by measuring the extent of bacterial growth inhibition resulting from the combination of different concentrations of the tested antibiotic and bacteriophage, using the “checkerboard” microdilution assay [53]. According to this method, the bacteriophage was titrated in 10-fold serial dilutions vertically across a 96-well microplate, while the antibiotic was titrated in 2-fold serial dilutions horizontally, after which bacterial culture was added to each well at a concentration of 10^8^ CFU/mL (0.5 McF). The plate was incubated at 37 °C for 24 h, after which the optical density was measured at 600 nm to determine bacterial culture growth.

Based on the obtained results, the fractional inhibitory concentration index (FICI) was calculated: FICI = (MIC APh/MIC A) + (MIC PhA/MIC Ph), where MIC APh is the minimum inhibitory concentration (MIC) of the antibiotic tested in combination with the phage, MIC A is the MIC of the antibiotic tested separately, MIC PhA is the MIC of the bacteriophage tested in combination with the antibiotic, and MIC Ph is the MIC of the bacteriophage tested separately (if it was impossible to determine the MIC of the bacteriophage due to the development of resistance, the maximum concentration of phage-containing samples—10^8^ PFU/mL—was used for the calculations). FICI ≤ 0.5 indicates a synergistic effect; 0.5 ˂ FICI ≤ 1 indicates an additive effect; 1 ˂ FICI ≤ 2 indicates no effect; FICI > 2 indicates an antagonistic effect.

### 2.16. Statistical Analysis and Graphical Representation of Results

Statistical analysis of the results was performed using Microsoft Excel. The Microsoft Office package was used to create tabular and graphical presentations of the results. For statistical reliability, all experiments were performed at least 3 times, the results were presented as the mean ± standard deviation (SD). Significant differences between the results of the experimental and control groups were determined using a two-tailed unpaired Student’s *t*-test and one-way analysis of variance (ANOVA) with Bonferroni post hoc test, with calculations performed in STATISTICA 12. A *p*-value ≤ 0.05 was considered statistically significant.

## 3. Results

### 3.1. Isolation of Bacteriophages, Morphological Characterization of Plaques and Viral Particles

From wastewater samples collected in the cities of Almaty, Shymkent, Konayev and Talgar, one bacteriophage isolate was obtained from each sample using the double-layer agar passage method. Bacteriophages were successfully isolated using three *E. coli* strains recovered from the feces of diarrheic calves: *E. coli* 4, *E. coli* 26, *E. coli* 46. The isolated phages were designated according to the standards of the International Committee on Taxonomy of Viruses (ICTV) (Table 1).

**Figure 1:**
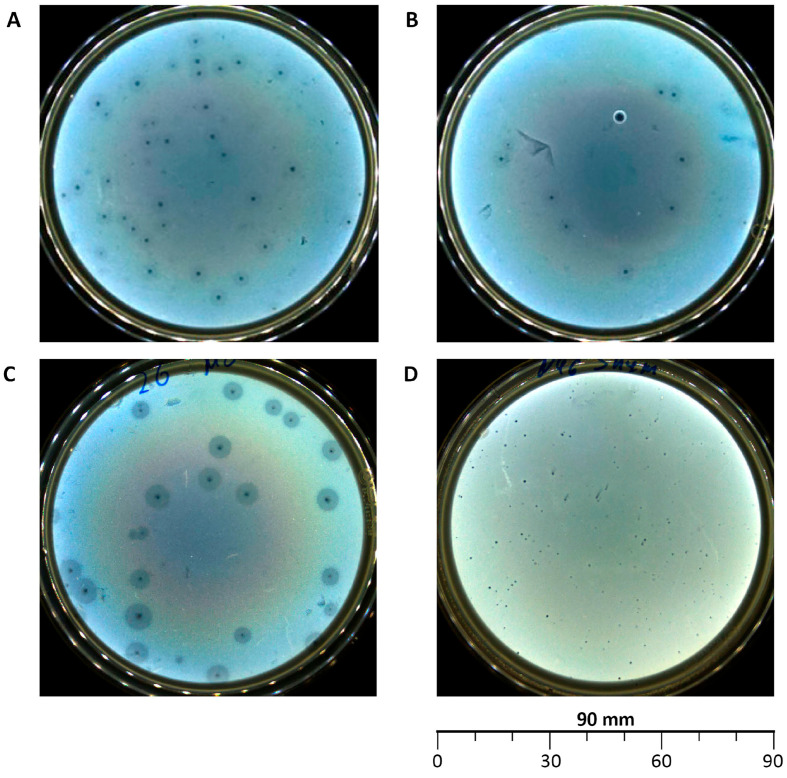
Morphology of plaques formed by the isolated bacteriophages: vB_EcoS_ABO/4 (**A**), vB_EcoM_PL/4 (**B**), vB_Eco_CWW/26 (**C**), vB_EcoM_ShWW/46 (**D**).

Plaques of phages vB_EcoS_ABO/4 and vB_EcoM_PL/4 had a round shape, 1–2 mm in diameter with a halo up to 5 mm (Figure 1A,B), plaques of vB_Eco_CWW/26 had a round shape with a diameter of 1–3 mm and a pronounced halo up to 8 mm (Figure 1C), and plaques of vB_EcoM_ShWW/46 were ≤1 mm in diameter, without a halo (Figure 1D).

**Figure 2:**
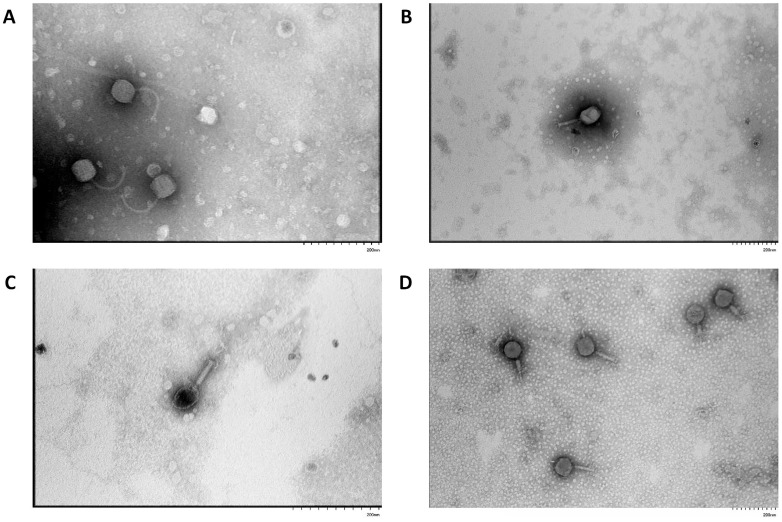
Electron microscopy of the vB_EcoS_ABO/4 (**A**), vB_EcoM_PL/4 (**B**), vB_Eco_CWW/26 (**C**) and vB_EcoM_ShWW/46 (**D**) bacteriophages.

Transmission electron microscopy revealed that the virions of bacteriophage vB_EcoS_ABO/4 exhibited a siphophage-like morphotype, characterized by a long, flexible, non-contractile tail approximately 160 nm in length and an icosahedral head with a diameter of about 60 nm (Figure 2A). In contrast, phages vB_EcoM_PL/4, vB_Eco_CWW/26, vB_EcoM_ShWW/46 possessed contractile tails approximately 100 nm in length and icosahedral heads measuring approximately 80–100 nm in diameter, which are typical features of a myophage-like morphotype (Figure 2B–D).

### 3.2. Phage Dynamics and Stability of Isolated Bacteriophages

Analysis of the multiplicity of infection showed that the maximum titer of viral particles was generated for phages vB_EcoS_ABO/4, vB_EcoM_PL/4, vB_Eco_CWW/26 and vB_EcoM_ShWW/46 at an infectious virus-to-bacterial cell ratio of 1:1 and was 8.2 × 10^8^, 4.9 × 10^8^, 2.9 × 10^8^ and 6.7 × 10^8^, respectively (Table 2). Thus, the optimal MOI for isolated bacteriophages was 1. The host bacterial strains used in the experiments correspond to the data in Table 1.

**Figure 3:**
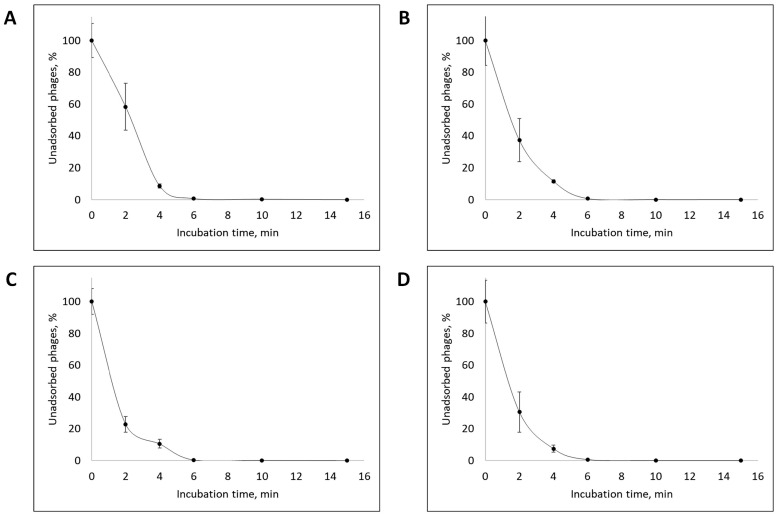
Adsorption rate of the vB_EcoS_ABO/4 (**A**), vB_EcoM_PL/4 (**B**), vB_Eco_CWW/26 (**C**) and vB_EcoM_ShWW/46 (**D**) bacteriophages. Results were presented as mean ± standard deviation (SD), *n* = 3. The initial concentration of all studied bacteriophages corresponded to an MOI of 1.

Adsorption rate analysis showed that approximately 90% of the viral particles of all isolated bacteriophages adsorbed to the host bacterial cells within the first 4 min, and adsorption of more than 99% of phage particles occurs within the first 10 min after the onset of phage infection (Figure 3).

**Figure 4:**
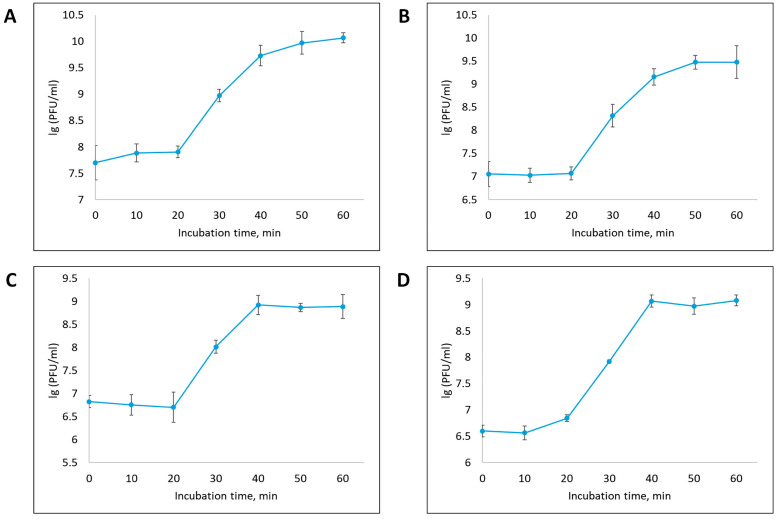
One-step growth curve of the vB_EcoS_ABO/4 (**A**), vB_EcoM_PL/4 (**B**), vB_Eco_CWW/26 (**C**) and vB_EcoM_ShWW/46 (**D**) bacteriophages. Results were presented as mean ± standard deviation (SD), *n* = 3. The initial concentration of all studied bacteriophages corresponded to an MOI of 0.1.

One-step growth curve analysis revealed that all isolated bacteriophages exhibited a latent period of approximately 20 min from the onset of phage infection. Upon completion of the latent period, an exponential increase in the number of phage particles was observed, which continued for the next 20 min (up to 40 min from the onset of infection), after which the phage titer stabilized, reaching a plateau (Figure 4). The size of the growth burst of phages vB_EcoS_ABO/4, vB_EcoM_PL/4, vB_Eco_CWW/26 and vB_EcoM_ShWW/46 was 127, 221, 135 and 225 PFU per cell, respectively.

**Figure 5:**
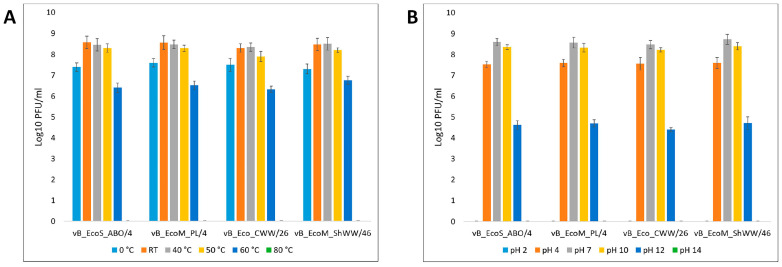
Titers of isolated bacteriophages at different temperatures (**A**) and pH (**B**) of the environment. Results were presented as mean ± standard deviation (SD), *n* = 3.

Thermal stability assessment showed that the isolated bacteriophages maintained lytic activity at a level of 10^8^ PFU/mL at room temperature, as well as after incubation at 40 °C and 50 °C. Freezing resulted in an average reduction of lytic phage titer by 1 log_10_, down to 10^7^ PFU/mL. At 60 °C, the titer further decreased to an average of 10^6^ PFU/mL, whereas incubation at 80 °C reduced lytic activity below the detection limit (Figure 5A).

The investigation of phage stability under different pH conditions revealed that, that at extreme pH values of 2 and pH 14, respectively, bacteriophages completely lost their lytic activity. Incubation at pH 4 reduced the phage titer on average to 10^7^ PFU/mL, whereas at pH 12 it decreased to 10^4^ PFU/mL. Incubation at pH 4 reduced the titer to an average of 10^7^ PFU/mL, and at pH 12 to 10^4^ PFU/mL. Under conditions of neutral (pH 7) and moderately alkaline environments (pH 10), the titer of bacteriophages remained at the initial level—about 10^8^ PFU/mL (Figure 5B).

Thus, the obtained data indicate that the studied bacteriophages have high resistance to temperature effects and pH values characteristic of the environment, without a significant decrease in lytic activity.

### 3.3. Level of Lytic Activity and Host Range of Isolated Bacteriophages

**Figure 6:**
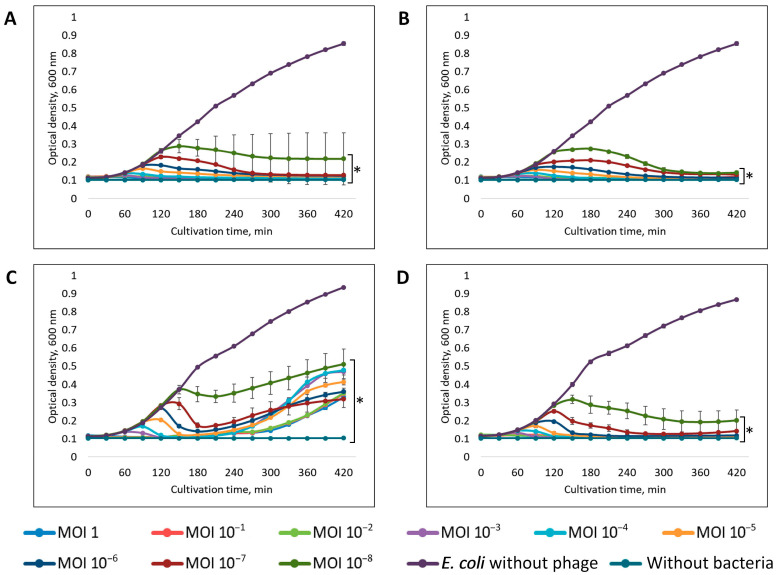
Level of lytic activity of the vB_EcoS_ABO/4 (**A**), vB_EcoM_PL/4 (**B**), vB_Eco_CWW/26 (**C**), vB_EcoM_ShWW/46 (**D**) bacteriophages. Results were presented as mean ± standard deviation (SD), *n* = 3. *p*-values are shown in comparison with the bacterial growth control group: * *p* ≤ 0.001.

Evaluation of lytic activity showed that the vB_EcoM_PL/4 bacteriophage demonstrated the highest efficiency, inhibiting host cell growth by more than 90% at all MOI values studied (Figure 6A). Phages vB_EcoS_ABO/4 and vB_EcoM_ShWW/46 inhibited the growth of host bacteria by more than 90% at MOI values from 1 to 10^−7^, at MOI values of 10^−8^ the growth of bacteria was suppressed by more than 80% (Figure 6B,D). The vB_Eco_CWW/26 phage exhibited minimal lytic activity, inhibiting the growth of the host bacteria by 40-95% within the first 3 h of incubation at MOI values ranging from 1 to 10^−8^. Further, the development of resistance was recorded in the *E. coli* 26 strain, which by the end of incubation led to the growth of the bacterial culture at a level of 25–50% of the control (Figure 6C).

The most rapid and pronounced inhibition of bacterial culture growth was observed at high MOI values (1 and 10^−1^): all the studied phages completely suppressed the growth of host bacteria within the first 30 min of cultivation. After an hour, the optical density of the suspension in the test samples, at these MOI values, decreased to a level comparable to the negative control.

To evaluate the lytic spectrum of the isolated bacteriophages, a sensitivity assay was performed using *E. coli* strains previously isolated from the feces of calves exhibiting signs of intestinal infection (Table S1).

According to the obtained results, phage vB_EcoS_ABO/4 exhibited the broadest lytic spectrum: it completely inhibited the growth (by 80–100%) of 11 strains and suppressed the growth of an additional 6 *E. coli* strains (Table 3). Phage vB_EcoM_ShWW/46 demonstrated the ability to inhibit the growth of 16 bacterial strains, but pronounced suppression (by 80–100%) was observed only in relation to three of them. Bacteriophage vB_EcoM_PL/4 showed lytic activity against 14 of the 35 studied strains of *E. coli*. At the same time, pronounced growth inhibition at the level of 80–100% was observed in 8 strains. Phage vB_Eco_CWW/26 was characterized by the narrowest spectrum of lytic activity, having the ability to inhibit the growth of only 11 of the 35 tested strains of *E. coli*.

### 3.4. Bioinformatic Analysis and Genomic Characterization of Isolated Bacteriophages

To analyze the structure and assess genetic diversity, full-genome sequences of four isolated bacteriophages were determined, after which a comparative analysis of the obtained results was carried out. Good quality of the initial data in the sequences for all phages was ensured in accordance with the FastQC parameters. Whole-genome sequencing revealed that bacteriophages vB_EcoS_ABO/4, vB_EcoM_PL/4, vB_EcoM_ShWW/46 possess linear double-stranded DNA genomes, whereas phage vB_Eco_CWW/26 contains a circular double-stranded DNA genome. The phage genome sizes ranged from 48,021 to 150,152 bp with GC content from 37.5% to 48.9%. The number of open reading frames (ORFs) increased with genome length: from 88 in phage vB_Eco_CWW/26 to 276 in phage vB_EcoM_ShWW/46. The presence of tRNA was detected only in two phages: vB_EcoM_PL/4 and vB_EcoM_ShWW/46. According to the results of the assembly completeness assessment, all the examined phage genomes demonstrated 100% completeness and were classified as high-quality, indicating a complete assembly without significant gaps or contamination, in accordance with the CheckV recommendations. Life cycle prediction performed using Bacphlip showed a high probability of the virulent type for all phages: from 85% for phage vB_Eco_CWW/26 to 93.7% for phage vB_EcoM_ShWW/46 (Table 4).

**Figure 7:**
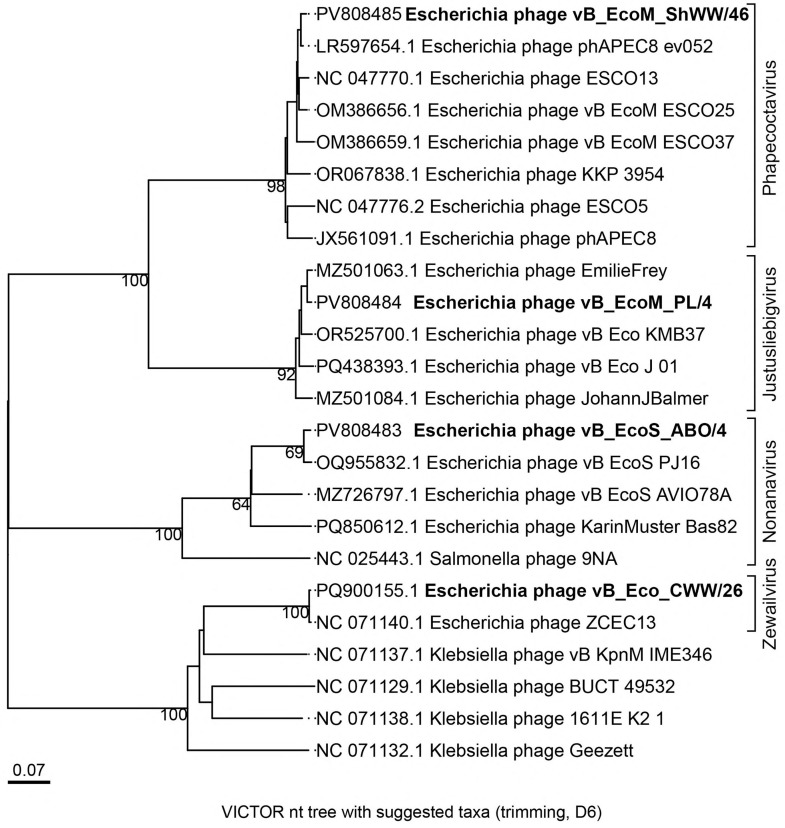
Phylogenetic tree generated by the VICTOR web service. All pairwise comparisons of the nucleotide sequences were conducted using the Genome-BLAST Distance Phylogeny (GBDP) method with the settings recommended for prokaryotic viruses.

To analyze the evolutionary relationships between the studied bacteriophages and other phages of the class *Caudoviricetes*, a phylogenetic tree was constructed using the Genome-BLAST Distance Phylogeny (GBDP) method. Figure 7 shows the GBDP phylogenetic tree constructed using the D6 protocol, which provided the highest degree of support with an adequate number of clusters at all taxonomic levels, indicating the best balance between resolution and tree stability. As a result, the genus-level classification of the studied bacteriophages was established. Phages vB_EcoM_PL/4 and vB_EcoM_ShWW/46 belonged to the same subfamily *Stephanstirmvirinae*, but to different genera, phage vB_EcoM_PL/4 was assigned to the *Justusliebigvirus* genus, and phage vB_EcoM_ShWW/46 to the *Phapecoctavirus* genus. Bacteriophage vB_Eco_CWW/26 showed a relationship with the *Escherichia* phage ZCEC13 (NC_071140.1), belonging to the *Jameshumphriesvirinae* subfamily, *Zewailvirus* genus. Whereas phage vB_EcoS_ABO/4 clustered in one clade with phages of the *Nonanavirus* genus.

Prediction of open reading frames using the standard genetic code in the genomes of phages vB_EcoS_ABO/4, vB_EcoM_PL/4, vB_Eco_CWW/26 and vB_EcoM_ShWW/46 allowed the identification of 98, 264, 88, 276 putative protein-coding genes, respectively.

**Figure 8:**
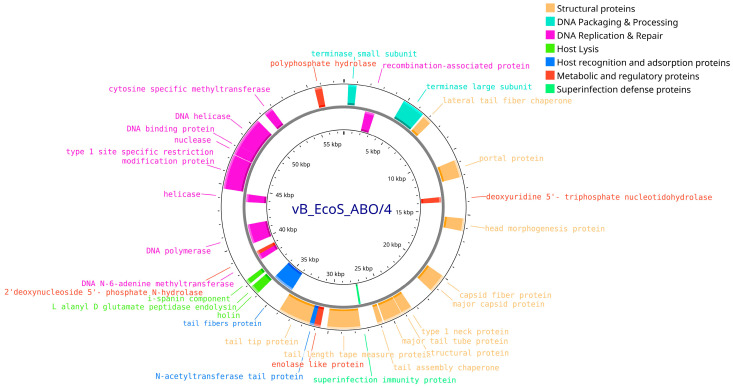
Whole-genome map of the vB_EcoS_ABO/4 bacteriophage. The map includes only proteins with assigned functions.

In the genome of phage vB_EcoS_ABO/4 (Accession no. PV808483), 33 of the 98 predicted proteins were functionally annotated and classified into the following categories: structural proteins, DNA packaging proteins, DNA replication and modification proteins, host recognition and adsorption proteins, host cell lysis proteins, metabolic and regulatory proteins, and proteins involved in superinfection immunity (Figure 8).

**Figure 9:**
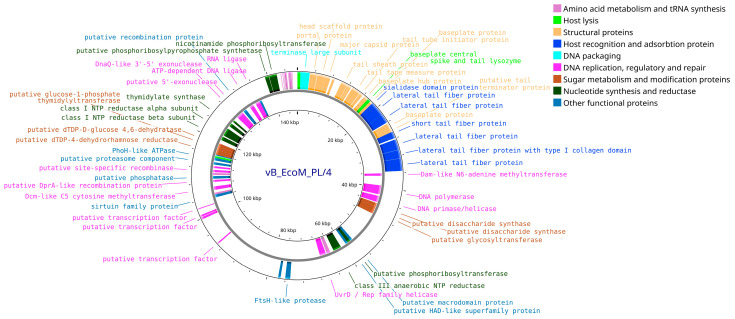
Whole-genome map of the vB_EcoM_PL/4 bacteriophage. The map includes only proteins with assigned functions.

Annotation of the vB_EcoM_PL/4 phage genome (Accession no. PV808484) resulted in the prediction of 264 coding sequences (CDS), of which 78 (29.6%) corresponded to proteins with established functions and 186 (70.4%) to hypothetical proteins with unidentified function. Among the functional CDS, 36 (46.2%) were located on the positive strand and 42 (53.8%) on the negative strand. The phage genome also contains 14 predicted tRNAs, including: tRNA-Ile, tRNA-Met, tRNA-Leu (×2), tRNA-Phe, tRNA-Pro, tRNA-Gln, tRNA-Gly, tRNA-Thr, tRNA-Asn, tRNA-Tyr, tRNA-Lys, tRNA-Ser (×2). In addition to functional proteins similar to those of the above-described phages (structural proteins, proteins of replication, packaging, lysis and interaction with the host cell), additional proteins associated with amino acid metabolism and tRNA biosynthesis, nucleotide synthesis, as well as enzymes involved in the metabolism and modification of sugars, including various reductases, were identified in the vB_EcoM_PL/4 phage genome (Figure 9).

**Figure 10:**
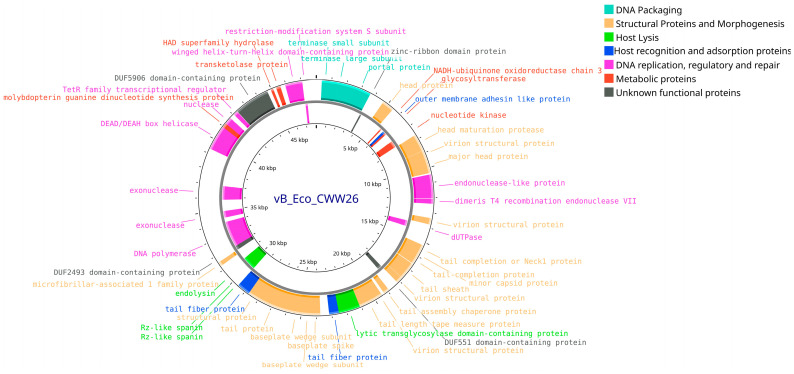
Whole-genome map of the vB_Eco_CWW26 bacteriophage. The map includes only proteins with assigned functions.

In the genome of phage vB_Eco_CWW/26 (Accession no. PQ900155.1), 88 putative CDS were annotated. Of these, 36 CDS (40.9%) were hypothetical proteins of unknown function, while 52 CDS (59.1%) encoded proteins with identified functions. Analysis revealed that 36 (69.3%) CDS with annotated functions were positioned on the leading strand, whereas 16 (30.7%) were located on the lagging strand. Among the proteins with known functions, structural components of the virion were identified, as well as proteins involved in phage genome replication, packaging, metabolic processes, host cell recognition and lysis (Figure 10).

**Figure 11:**
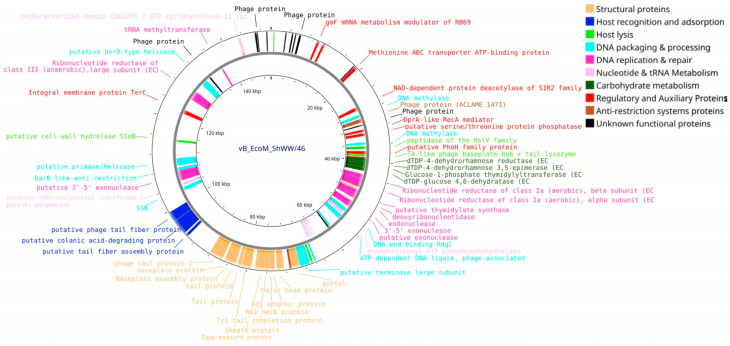
Whole-genome map of the vB_EcoM_ShWW/46 bacteriophage. The map includes only proteins with assigned functions.

Genomic analysis of the vB_EcoM_ShWW/46 phage (Accession no. PV808485) revealed 276 putative protein-CDS, of which 75 (27.2%) are proteins with an established function and 201 (72.8%) are hypothetical proteins with an unidentified function. Among them, the functional CDS 40 were located on the leading DNA strand and 35 on the lagging strand. The functional proteins include structural components of the virion, proteins involved in the processes of DNA replication, packaging and repair, proteins providing recognition and lysis of the host cell, as well as proteins involved in nucleotide, amino acid and carbohydrate metabolism. In addition, regulatory proteins and proteins putatively involved in the anti-restriction system were identified. Using the tRNAscan-SE program, 10 tRNAs were identified in the genome: tRNA-Met (×2), tRNA-Ile, tRNA-Pro, tRNA-Gln, tRNA-Gly, tRNA-Thr, tRNA-Asn, tRNA-Ser and tRNA-Arg (Figure 11).

### 3.5. Assessment of the Synergistic Interaction Between Isolated Bacteriophages and Antibiotics

To evaluate the synergistic effect of the combined use of bacteriophages and antibacterial agents, three antibiotics commonly used in veterinary practice and exhibiting varying levels of resistance among the *E. coli* strains included in this study were selected (Table S1). Ampicillin (AMP) showed the narrowest range of antibacterial activity, with 20 of the 35 tested *E. coli* strains demonstrating resistance. Gentamicin (GEN), representing an intermediate spectrum, was ineffective against 11 strains. Colistin (COL) demonstrated the broadest spectrum of activity, with only one *E. coli* strain displaying resistance.

A cocktail containing equal volumes of all four isolated bacteriophages, each with a titer of ≈10^8^ PFU/mL, was used as a phage-containing sample.

As bacterial test cultures, *E. coli* strains that were not the primary hosts of the isolated phages and exhibited varying levels of resistance to antibacterial agents were selected. *E. coli* 1MC is a strain sensitive to all studied bacteriophages and antibiotics. *E. coli* 23-2Y is a strain sensitive to 7 of 8 used antibiotics and to phages vB_EcoS_ABO/4, vB_EcoM_PL/4. *E. coli* 17-1YF is a strain with moderate resistance, sensitive to 5 antibiotics and phages vB_EcoS_ABO/4, vB_EcoM_PL/4, vB_EcoM_ShWW/46. *E. coli* 32 is a strain with pronounced resistance, insensitive to all isolated bacteriophages and resistant to 6 of 8 tested antibiotics. *E. coli* 35 is a multiresistant strain demonstrating complete resistance to all studied bacteriophages and antibacterial drugs [9].

**Figure 12:**
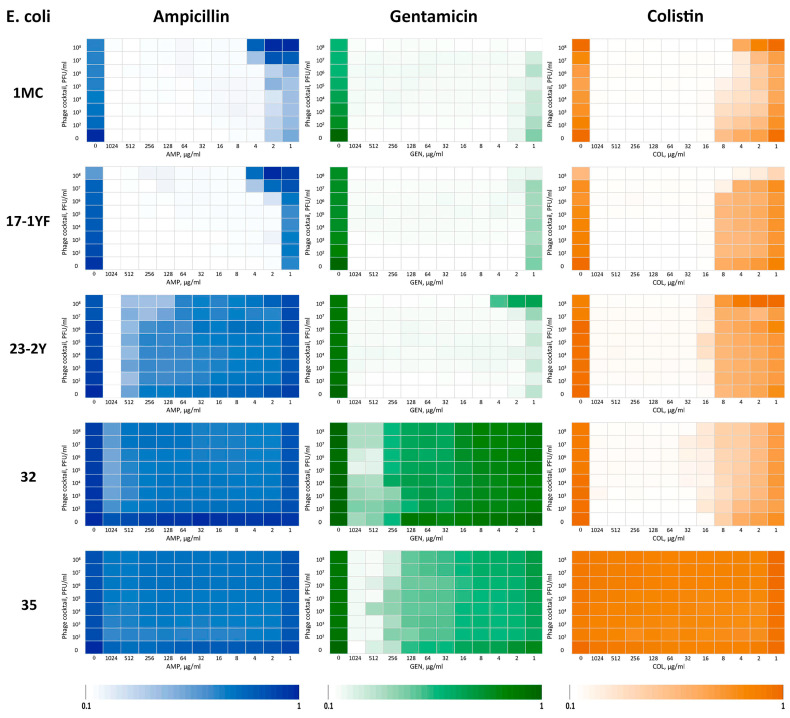
Analysis of the combined effect of the phage cocktail and antibiotics using the “checkerboard” method.

Based on the results of the experiments and calculation of the FICI index, it was found (Table 5, Figure 12) that with the combined use of AMP and phage cocktail on two *E. coli* strains (1MC and 17-1YF), a weak additive effect was observed, on two more *E. coli* strains (32 and 35) the interaction effect was absent, and on strain *E. coli* 23-2Y a synergistic effect was noted. When using the phage cocktail with GEN on four strains, a weak synergistic effect was shown (1MC, 17-1YF, 32, 35), on strain *E. coli* 23-2Y—a weak additive effect. The combined use of COL and the cocktail showed a pronounced synergistic effect on strains 1MC and 17-1YF, a weak synergistic effect on *E. coli* strain 32, a weak additive effect on strain 23-2Y, and no interaction effect on *E. coli* 35 strain.

Thus, when analyzing the interaction of a phage cocktail with three antibiotics against five strains of *E. coli*, an additive effect was observed in 4 cases, no effect in 3 cases, and a synergistic effect in 8 cases, accounting for more than half of the tested combinations.

## 4. Discussion

The widespread spread of multiple drug resistance among pathogenic microorganisms is one of the major problems of modern veterinary medicine. The rate of resistance development is especially high in animal husbandry due to the unreasonable and uncontrolled use of antibacterial drugs. In addition, the risk of the spread of resistant bacterial infections is aggravated by a decrease in the effectiveness of antibiotics used in practice, as well as a sharp slowdown in the rate of development and implementation of new antibacterial preparations [54,55,56,57]. In the current circumstances, there is a need to introduce new methods of antibacterial therapy or improve existing practices. For this reason, in recent decades, interest in bacteriophages and phage therapy as an alternative to antibiotic therapy has sharply increased [58,59,60,61]. Therefore, the goal of our research was to isolate and study new bacteriophages capable of lysing pathogenic forms of *E. coli*, as well as to study the antibacterial activity of isolated phages in combination with antibiotics.

As a result of the study, four bacteriophages capable of lysing potential causative agents of calf colibacillosis were isolated from wastewater collected in various cities of Kazakhstan (Almaty, Shymkent, Konayev, Talgar), using three *E. coli* strains. The findings highlight the high phage diversity present in wastewater and high probability of targeted isolation of lytically active bacteriophages [62]. In accordance with ICTV guidelines, the isolated phages were designated as vB_EcoS_ABO/4, vB_EcoM_PL/4, vB_Eco_CWW/26 and vB_EcoM_ShWW/46.

Phenotypic characterization of the plaques revealed the presence of faint halos in phages vB_EcoS_ABO/4, vB_EcoM_PL/4 and a pronounced halo in phage vB_Eco_CWW/26, which may indicate the ability of these phages to diffuse, as well as the presence of bacteriolysins [63]. It is known that the presence of bacteriolysins, capable of decomposing bacterial polysaccharides, increases the efficiency of phage infection, since they promote the destruction of bacterial walls and biofilms [64]. This, in turn, facilitates the access of bacteriophages, components of the immune system and antibacterial drugs to target cells [65].

Transmission electron microscopy allowed the classification of the isolated phages by morphotype. Phage vB_EcoS_ABO/4 was assigned to the sipho-like group due to its icosahedral head and long, flexible, non-contractile tail. In contrast, the other three phages (vB_EcoM_PL/4, vB_Eco_CWW/26, vB_EcoM_ShWW/46) exhibited typical features of myo-like morphology, including a contractile tail, baseplate with spikes, and tail fibers. The structural characteristics of the isolated bacteriophage virions suggest their preliminary classification within the class *Caudoviricetes*. Both siphovirus- and myovirus-like phages of the *Caudoviricetes* class are typical representatives of bacteriophages infecting *E. coli*. [66].

Evaluation of the multiplicity of infection showed that the maximum number of lytically active phage particles (2.9–8.2 × 10^8^ PFU/mL) is formed at an infectious ratio of 1:1 (MOI 1), i.e., with an equal number of phages and bacterial cells. It should be noted that at MOI values of 10, isolated phages also showed high titers (1.6–5.5 × 10^8^ PFU/mL) comparable to titers at MOI 1, which may indicate high efficiency of phage replication even with an excess of viral particles at the infection stage.

All isolated bacteriophages exhibited a high adsorption rate, with approximately 90% of viral particles attaching to host cells within the first 4 min, indicating efficient initial phage–host cell interaction [67].

One-step growth curve analysis revealed that the isolated bacteriophages possess a short latent period (20 min) followed by an active replication phase completed by 40th minute. The burst size ranged from 127 to 225 phages per cell. These results confirm the high replication rate of the studied phages and their capacity for rapid host cell lysis and efficient propagation [68]. Bacteriophages with a short latent period and high productivity are considered the most promising for use in phage therapy, since they are capable of destroying pathogenic bacteria in a short time and reducing the likelihood of the formation of resistant clones.

Thermal stability testing demonstrated that all isolated bacteriophages maintained their initial lytic titers within a temperature range from room temperature up to 50 °C. Freezing the samples without cryoprotectants led to an average reduction in phage titer by one order; however, lytic activity remained at an acceptable level. Therefore, the isolated bacteriophages can be effectively stored and used under standard conditions, and can also be frozen for long-term storage with only minimal impact on efficacy. Such resistance to temperature effects makes isolated bacteriophages promising candidates for practical use, allowing for simplified storage and transportation conditions, as well as reducing logistics costs.

Assessment of pH stability showed that the isolated bacteriophages retained high lytic activity within the physiologically relevant pH range. Maximum titers (~10^8^ PFU/mL) were observed at neutral pH 7.0 and moderately alkaline pH 10.0, confirming their suitability for use in conditions close to normal physiological parameters, including systemic and oral therapy. At pH 4.0, a moderate decrease in titer to ~10^7^ PFU/mL was observed, probably associated with partial inactivation of virions, but lytic activity remained at a therapeutically significant level. At pH 12, the titer decreased to ~10^4^ PFU/mL, indicating significant damage to viral particles under strongly alkaline conditions. At extreme pH values of pH 2.0 and pH 14.0, lytic activity was completely lost, probably due to capsid destruction or protein denaturation. These results are consistent with the literature data, according to which most phages lose activity at pH below 3.0 or above 12.0 [69].

Stability in the pH range of 4.0–10.0 makes the studied phages promising for practical application. The use of encapsulation or buffer systems can further increase their stability during oral administration [70,71]. In addition, the retention of activity at pH 10 indicates the potential effectiveness of phages in conditions of sanitization of livestock facilities using alkaline detergents.

The results of determining the lytic activity of isolated bacteriophages showed that the most effective phage was vB_EcoM_PL/4, which inhibited bacterial growth by more than 90% at all MOI values studied. This indicates its high virulence. Phages vB_EcoS_ABO/4 and vB_EcoM_ShWW/46 also showed high lytic activity, inhibiting the growth of host bacteria at MOI values from 1 to 10^−7^ by more than 90%. At the lowest concentration studied (MOI 10^−8^), the lytic activity of these phages decreased, but remained at the level of 80% inhibition of the growth of host bacterial cultures, which indicates their ability to initiate infection even at low concentrations of viral particles. Such properties are especially important in conditions where achieving high phage doses is difficult, for example, during oral delivery or use in complex biological environments [72].

Phage vB_Eco_CWW/26 exhibited the lowest lytic activity. Despite initially demonstrating a high level of bacterial growth inhibition, by the end of the incubation period, regrowth of the bacterial culture was observed (up to 25–50% of the control). This is likely due to the rapid adaptation of bacteria and the development of resistance to the phage [73]. Moreover, as shown in Figure 6C, high phage concentrations accelerate the rate of lysis, thereby promoting the rapid selection of resistant bacterial clones, which is reflected by a sharp increase in bacterial cell growth. In contrast, at low phage concentrations, a relatively gradual increase in bacterial growth was observed.

In the case of bacteriophages vB_EcoM_PL/4, vB_EcoS_ABO/4 and vB_EcoM_ShWW/46, secondary growth of bacterial cells was not recorded, which is probably due to the insufficient duration of incubation for the formation of phage resistance mechanisms in bacterial cultures.

Such results highlight the need for a comprehensive study of newly isolated phages, as well as the possibility of using combination therapy strategies, such as the use of phage cocktails or the combined use of phages and antibiotics.

The host range analysis of the isolated bacteriophages demonstrated that phage vB_EcoS_ABO/4 exhibited the broadest lytic spectrum, completely inhibiting the growth of 11 out of 35 *E. coli* strains and suppressing the growth of an additional 6 strains. These characteristics make it a more universal and effective candidate for the development of phage-based therapeutic preparations. Phage vB_EcoM_ShWW/46 was active against 16 *E. coli* strains, but only in three cases was the bacterial culture growth inhibited by 80–100%, which may indicate a bacteriostatic effect or the presence of partial resistance mechanisms to this phage in the most sensitive *E. coli* strains. Phage vB_EcoM_PL/4 demonstrated activity against 14 *E. coli* strains, with growth inhibited by 80–100% in eight of them. Phage vB_Eco_CWW/26 demonstrated the narrowest host range, effectively inhibiting the growth of only 11 of 35 *E. coli* strains. Despite their rather narrow specificity, phages vB_EcoM_PL/4 and vB_Eco_CWW/26 can be successfully used in targeted therapy against sensitive strains, and can also be included in phage cocktails, increasing their effectiveness and spectrum of lytic activity [74].

Thus, the differences in the lytic activity spectrum among the studied phages highlight the necessity of their combined use in the form of cocktails. This will not only expand the coverage of pathogens, but also reduce the risk of developing phage resistance, which is especially important in the treatment of infections caused by genetically heterogeneous populations of *E. coli* [75].

The phylogenetic analysis performed using the Genome-BLAST Distance Phylogeny (GBDP) method allowed us to clarify the taxonomic position of the studied bacteriophages within the *Caudoviricetes* class. The data obtained indicate the phylogenetic diversity of phages, despite their potential similarity in host or habitat.

Phages vB_EcoM_PL/4 and vB_EcoM_ShWW/46, clustered within the *Stephanstirmvirinae* subfamily, were nevertheless assigned to different genera, *Justusliebigvirus* and *Phapecoctavirus*, respectively. This indicates the presence of both common evolutionary features characteristic of the subfamily and specific genomic differences sufficient for separation at the genus level. This result highlights the importance of high-resolution analysis methods in phage classification, especially within closely related taxa. Phages vB_Eco_CWW26 and vB_EcoS_ABO/4 showed phylogenetic relationships with representatives of the *Zewailvirus* and *Nonanavirus* genera, respectively. Despite the lack of obvious phylogenetic similarity with other phages studied, phages vB_Eco_CWW/26 and vB_EcoS_ABO/4 maintain relationships with representatives of the same class, demonstrating a high degree of genetic diversity characteristic of bacteriophages even within the same taxonomic level. Thus, the results of the present study also confirm the taxonomic heterogeneity of bacteriophages and emphasize the importance of whole genome analysis for the correct classification of phages. The identified phylogenetic relationships can serve as a basis for further studies on the evolution and functional diversity of phages within the *Caudoviricetes* class.

Annotation and comparative analysis of the genomes of phages vB_EcoS_ABO/4, vB_EcoM_PL/4, vB_Eco_CWW/26 and vB_EcoM_ShWW/46 revealed both common and unique features, reflecting the diversity and adaptability of bacteriophages to different environments and host organisms. The number of predicted CDS varied across the four genomes, ranging from 88 (vB_Eco_CWW/26) to 276 (vB_EcoM_ShWW/46). The proportion of proteins with predicted functions ranged from 27.2% (vB_EcoM_ShWW/46) to 59.1% (vB_Eco_CWW/26), reflecting the potential presence of new, as yet uncharacterized proteins. The high percentage of putative proteins, especially in phage vB_EcoM_ShWW/46, highlights the need for further functional studies, including transcriptomics and proteomics [76]. All phage genomes contained key groups of proteins essential for the viral life cycle: virion structural proteins, replication proteins, DNA packaging proteins, and host cell lysis proteins. These components represent the “conserved core” of phage genomes, which is necessary for successful infection and spread [77]. The greater number of genes encoding proteins involved in the processes of DNA replication, repair, and transcription in the genomes of phages vB_EcoM_ShWW/46 and vB_EcoM_PL/4 probably reflects their evolutionary features and may help them adapt to conditions in which the expression or availability of cellular enzymes is limited (e.g., under stress or bacterial latency) or allow them to compete in multispecies communities (e.g., in the intestine or soil), where bacterial cells are often infected by several phages simultaneously. Phages with a more complete set of their own enzymes gain an advantage because they can reproduce faster and more efficiently without competing for cellular resources [78,79].

Additionally, the genomes of the studied phages contained metabolic and regulatory proteins. In particular, the genomes of phages vB_EcoM_PL/4 and vB_EcoM_ShWW/46 encoded proteins involved in amino acid metabolism, tRNA biosynthesis, and nucleotide and sugar synthesis, including reductases. This suggests the ability of the phages to modulate host cell metabolism to enhance replication efficiency under varying environmental conditions [80].

Analysis of genomes using the tRNAscan-SE program revealed the presence of tRNA in the genomes of phages vB_EcoM_PL/4 and vB_EcoM_ShWW/46, while phages vB_EcoS_ABO/4 and vB_Eco_CWW/26 did not contain tRNA. The absence of tRNA in the phage genomes suggests a high degree of their dependence on the host cell translation apparatus. This may reflect their evolutionary adaptation to the efficient use of coding and translational resources of the bacterium. In turn, the presence of tRNA in the bacteriophage genome may indicate a strategy for optimizing the expression of their own genes, in particular under conditions of codon mismatch between the phage genome and the host’s preferences [81,82,83].

Analysis of whole genome sequences using the PhageLeads tool revealed that the genomes of all studied phages lack gene sequences encoding integrase, recombinase, and repressors—key markers of lysogenic viruses—as well as genes associated with antibiotic resistance and bacterial virulence. Therefore, the isolated bacteriophages can be considered safe candidates for use as phage therapy agents.

Thus, the conducted analysis allows us to state that although phages vB_EcoS_ABO/4, vB_EcoM_PL/4, vB_Eco_CWW/26 and vB_EcoM_ShWW/46 share a common set of key genes that ensure the reproductive cycle, each of them also has unique functional features. These differences may be associated with adaptation to specific bacterial hosts, ecological niche or strategies to evade cellular defenses. The obtained data expand our understanding of the genomic organization and evolutionary plasticity of bacteriophages and highlight the potential for further study of their biotechnological applications.

Phage therapy offers several advantages, including high specificity, which allows the elimination of pathogenic bacteria without harming the natural microbiota, the ability to self-replicate at the site of infection, and natural clearance from the body after the pathogen is eliminated. Moreover, due to their natural origin, phages are considered virtually safe for animals [84]. Significant limitations of phage therapy include the high probability of developing resistance mechanisms in target bacteria, as well as the development of immune reactions following the administration of phage preparations, which can cause either allergic reactions or the formation of specific antibodies that neutralize phage virions, thereby reducing or nullifying the therapeutic efficacy [85].

Considering both the advantages and limitations of phage therapy, as well as the long-standing experience with antibiotic use, the concept of combined phage–antibiotic therapy was proposed. Research conducted in this direction has shown that the simultaneous use of antibiotics and bacteriophages leads to more effective and rapid eradication of antibiotic-resistant forms of pathogenic bacteria than when using one of these methods [86,87,88,89,90].

Therefore, in the present study, in addition to the isolation and characterization of novel bacteriophages capable of lysing potential causative agents of calf colibacillosis, experiments were conducted to evaluate the synergistic effect of the combined use of a cocktail based on the isolated phages and three antibiotics (ampicillin, gentamicin, and colistin) commonly used in veterinary practice and exhibiting varying levels of resistance among *E. coli* strains.

Synergy between phages and antibiotics was assessed using the “checkerboard” method with MIC determination and FICI calculation. However, this approach has limitations when applied to phage therapy, since MIC and FICI reflect only static endpoints (usually after 24 h of incubation), whereas bacteriophages are dynamic biological agents whose lytic and reproductive activity depends on many factors (adsorption rate, target cell sensitivity, development of phage resistance, influence of environmental conditions, etc.). As a result, the same final MIC value may correspond to fundamentally different trajectories of dynamics. Moreover, due to the emergence of phage-resistant clones at the end of incubation, the samples may exhibit bacterial growth, despite the pronounced growth suppression observed at the early stages, which is also supported by the data obtained in our study.

Nevertheless, despite the above limitations, the “checkerboard” method turned out to be the most optimal in our conditions, since the studies were conducted using three antibiotics and five test cultures of bacteria in a wide range of antibiotic concentrations (1–1024 μg/mL) and phage titers (10^2^–10^8^ PFU/mL), which corresponded to 1440 experimental variants and 4320 tests, taking into account triplicates. Under these conditions, the method provided high reproducibility and, when generating heatmaps, enabled clear visualization of the “synergy windows” depending on the combination of antibiotic doses and bacteriophage titers.

The most pronounced interaction was observed with the combination of the phage cocktail and colistin, which provided a synergistic effect against 3 of the 5 *E. coli* strains tested, including two cases of pronounced synergy. When combined with gentamicin, 4 strains showed a weak synergistic effect, and one strain showed a weak additive effect. The use of ampicillin in combination with the cocktail demonstrated a predominantly weak additive effect (two cases) or no interaction (two cases), with the exception of one case of synergy.

Differences in the manifestation of the synergistic effect during the combined use of a phage cocktail with antibiotics may be due not only to the individual characteristics of the bacterial strains, but also to differences in the mechanisms of action of the antibiotics used. Thus, ampicillin is an antibiotic from the β-lactam group, the mechanism of action of which is based on the inhibition of the synthesis of peptide glycan, the main component of the bacterial cell wall. Indirectly, phage infection can disrupt the synthesis of β-lactamases, thereby weakening the protective mechanisms of bacterial cells [91]. However, to date there is no convincing data confirming direct inhibition of β-lactamase synthesis by natural phages. In this regard, it can be assumed that a pronounced synergistic effect in the combined use of phages and β-lactam antibiotics is not always observed and depends on the characteristics of the interaction of specific bacterial strains and phage [92,93]. This is consistent with our findings, which demonstrated that the combination of ampicillin with the phage cocktail predominantly resulted in weak additive effects or no interaction.

The action of gentamicin is based on binding to the 30S subunit of the bacterial ribosome and disruption of protein synthesis processes. Since many mechanisms of bacterial resistance to phages—such as restriction-modification systems (R-M systems), CRISPR-Cas, phage-inhibitor proteins (Phage Inhibitor Proteins), Bacteriophage Exclusion (BREX) and Defense Island System Associated with Restriction-Modification (DISARM) systems—directly depend on the expression of the corresponding proteins, the action of gentamicin can contribute to their suppression and increase the susceptibility of bacterial cells to phage infection [94,95,96,97]. A similar effect was likely observed in our experiments, where the combined application of gentamicin and the investigated phages resulted in a weak synergistic effect in 4 out of 5 *E. coli* strains, and a weak additive effect in one strain.

The mechanism of action of colistin is based on the disruption of the outer membrane integrity of Gram-negative bacteria, which leads to increased permeability of their cells. Presumably, an increase in the permeability of the outer membranes of Gram-negative bacteria facilitates the penetration of bacteriophages, which can enhance the effectiveness of phage infection and, as a result, accelerate the lysis of bacterial cells [98]. This assumption is indirectly confirmed by the results of our studies, which showed that with the combined use of colistin with the studied bacteriophages, a synergistic effect was observed in relation to 3 of 5 strains of *E. coli*.

A generalized analysis of the experimental results assessing the synergistic effect of the interaction between the studied bacteriophages and antibiotics showed that out of 15 tested combinations (3 antibiotics × 5 strains), a synergistic effect was detected in 8 cases (53.3%), an additive effect in 4 cases (26.7%), and no interaction in 3 cases (20%). The data obtained show that the combined use of bacteriophages and traditional antibiotics can provide a more pronounced inhibitory effect against *E. coli* than their use separately.

Moreover, the results obtained from the synergy assessment experiments showed that bacterial test cultures developed resistance to all isolated bacteriophages after 24 h of incubation (Figure 12). The same results also revealed that, in certain experimental variants (Ampicillin—*E. coli* 1MC, 17-1YF; Gentamicin—*E. coli* 23-2Y; Colistin—*E. coli* 1MC, 23-2Y) and at the same antibiotic concentration, the growth of the bacterial test culture was higher at higher phage concentrations compared to lower phage concentrations. This is apparently due to accelerated phage lysis and, as a consequence, accelerated selection of more resistant strains [99,100]. Such observations highlight the need for careful selection of phage concentrations when developing phage-based therapeutics and underscore the relevance of combination therapy strategies, especially in the presence of heterogeneous bacterial populations.

Thus, the combined use of bacteriophages and antibiotics, especially in cases of partially reduced sensitivity of pathogens to the latter, is a promising approach that can enhance the therapeutic effect, reduce the required dose of antibiotics and overcome the resistance of bacterial pathogens.

## 5. Conclusions

As a result of the work carried out, 4 bacteriophages with the ability to lyse potential pathogens of calf colibacillosis were isolated from wastewater from various cities in Kazakhstan. The results of the study on phage dynamics and biological properties demonstrated that the isolated phages exhibit high stability under standard environmental conditions, rapid adsorption and replication, pronounced lytic activity, and a broad host range. The whole-genome sequencing and the study of the virion structure made it possible to establish that the isolated bacteriophages are representatives of four different genera of the *Caudoviricetes* class, and their genomes did not contain genes encoding factors of lysogeny, bacterial resistance to antibiotics, or bacterial virulence.

Evaluation of the interaction during the combined use of the studied bacteriophages and traditional antibiotics against *E. coli* strains showed that a synergistic effect was observed in more than 50% of cases, and an additive effect in another 27% of cases.

Thus, the in vitro studies showed that the isolated bacteriophages are promising candidates for the development of effective and safe antibacterial agents both as independent phage preparations and as part of combination regimens with traditional antibiotics. However, to confirm their therapeutic efficacy in the treatment of infections caused by calf colibacillosis pathogens, further in vivo studies are required.

## Figures and Tables

**Table 1:** Designation of isolated bacteriophages.

Designation of Isolated Bacteriophage	Bacterial Host Strain	City (Source of Phage-Containing Wastewater)
vB_EcoS_ABO/4	*E. coli* 4	Almaty
vB_EcoM_PL/4		Talgar
vB_Eco_CWW/26	*E. coli* 26	Konayev
vB_EcoM_ShWW/46	*E. coli* 46	Shymkent

**Table 2:** Titer of isolated bacteriophages at different MOI values.

Bacteriophage	MOI	Number PFU	Bacteriophage	MOI	Number PFU
vB_EcoS_ABO/4	10^−6^	98 ± 15 *	vB_EcoM_PL/4	10^−6^	38 ± 6
	10^−5^	8.2 ± 0.4 × 10^2^		10^−5^	3.6 ± 0.4 × 10^2^
	10^−4^	4.7 ± 0.3 × 10^4^		10^−4^	2.7 ± 0.1 × 10^4^
	10^−3^	5.1 ± 0.3 × 10^5^		10^−3^	4.1 ± 0.2 × 10^5^
	10^−2^	2.5 ± 0.1 × 10^7^		10^−2^	3.8 ± 0.1 × 10^7^
	10^−1^	4.1 ± 0.2 × 10^8^		10^−1^	7.5 ± 0.2 × 10^7^
	1	8.2 ± 0.3 × 10^8^		1	4.9 ± 0.4 × 10^8^
	10	5.5 ± 0.3 × 10^8^		10	2.1 ± 0.1 × 10^8^
vB_Eco_CWW/26	10^−6^	13 ± 4	vB_EcoM_ShWW/46	10^−6^	54 ± 9
	10^−5^	51 ± 11		10^−5^	5.6 ± 0.4 × 10^2^
	10^−4^	2.7 ± 0.2 × 10^4^		10^−4^	2.7 ± 0.1 × 10^4^
	10^−3^	4.8 ± 0.3 × 10^5^		10^−3^	4.5 ± 0.3 × 10^5^
	10^−2^	3.2 ± 0.2 × 10^7^		10^−2^	3.2 ± 0.1 × 10^7^
	10^−1^	6.4 ± 0.2 × 10^7^		10^−1^	8.6 ± 0.4 × 10^7^
	1	2.9 ± 0.1 × 10^8^		1	6.7 ± 0.3 × 10^8^
	10	1.6 ± 0.1 × 10^8^		10	3.3 ± 0.1 × 10^8^

* Results were presented as mean ± standard deviation (SD), *n* = 3.

**Table 3:** Host range of isolated *E. coli* bacteriophages.

№	*E. coli* Strains	Bacteriophages			
		vB_EcoS_ABO/4	vB_EcoM_PL/4	vB_Eco_CWW/26	vB_EcoM_ShWW/46
1	1MC	+++*	+++	++	+++
2	4MY	+++	+++	+++	—
3	8MY	++	—	—	+
4	9MY	—	—	+	—
5	12MC	++	—	—	+
6	16-1ML	—	—	—	—
7	16-1P	++	++	++	—
8	16-2CF	—	—	++	—
9	17-1YF	+	+	—	++
10	18 YML	+++	+++	+++	—
11	19-AY	—	—	++	—
12	19-BYF	—	—	—	+
13	20-1 YF	—	—	—	—
14	20-2 Y	—	—	—	—
15	21-1C	—	—	—	—
16	21-2YF	+++	+	—	+
17	22-1YF	++	+	—	++
18	23-1YF	—	—	—	++
19	23-2Y	++	+	—	—
20	24CF	—	—	++	+
21	25-1CF	—	—	++	—
22	25-2 P	+++	+	—	—
23	25-2W	+++	—	—	+
24	26	—	—	+++	+
25	27	—	—	+	—
26	32	—	—	—	—
27	33	—	—	—	—
28	35	—	—	—	—
29	37	—	—	—	—
30	38	—	—	—	++
31	39	+++	+++	—	+
32	44	+++	+++	—	+++
33	45	+++	+++	—	—
34	46	+++	+++	—	+++
35	48	+++	+++	—	++
Spectrum Index		17	14	11	16

*+++ - inhibition of bacterial culture growth by 80–100%; ++ - inhibition of bacterial culture growth by 50–80%; + - inhibition of bacterial culture growth by 20–50%; — No inhibition of bacterial culture growth. 100% inhibition of bacterial growth—optical density value corresponding to samples without addition of bacterial suspension (negative control), 0% inhibition of bacterial growth is the optical density value corresponding to samples without the addition of phage lysates (positive control).

**Table 4:** Genomic details of the studied bacteriophages.

Feature	vB_Eco_CWW/26	vB_EcoS_ABO/4	vB_EcoM_PL/4	vB_EcoM_ShWW/46
Genome size, bp	48,021	57,756	146,666	150,152
Number of contig N50	1	1	1	1
N50 length (bp)	48,021	57,756	146,666	150,152
CheckV completeness (%)	100	100	100	100
CheckV quality	High-quality	High-quality	High-quality	High-quality
ORF number	88	98	264	276
GC content (%)	48.9	43.5	37.5	39
tRNAs	-	-	14	10
Bacphlip life cycle (%)	Virulent (85)	Virulent (91.4)	Virulent (91.2)	Virulent (93.7)

**Table 5:** Fractional inhibitory concentration index (FICI) for the combined use of isolated bacteriophages and antibiotics.

*E. coli*	FICI		
	AMP	GEN	COL
1MC	1 *	0.5	0.13
17-1YF	1	0.5	0.12
23-2Y	0.25	1	1
32	2	0.5	0.5
35	2	0.5	2

* FICI ≤ 0.5 indicates a synergistic effect; 0.5 ˂ FICI ≤ 1 indicates an additive effect; 1 ˂ FICI ≤ 2 indicates no effect; FICI > 2 indicates an antagonistic effect.

## Data Availability

The genome sequences were deposited in GenBank under the accession numbers: PQ900155.1, PV808483, PV808484, PV808485. The raw sequencing reads were submitted to the NCBI SRA under the accession numbers: SRR35018643, SRR35023002, SRR35024783, SRR35026102.

## Supplementary Materials

The following supporting information can be downloaded at: https://www.mdpi.com/article/10.3390/vetsci12090817/s1, Table S1: Antibiotic susceptibility of *E. coli* strains. Table S2: Interpretation breakpoints of the various antimicrobial agents used in the study.

## Abbreviations

The following abbreviations are used in this manuscript:

NB	Nutrient Broth
NA	Nutrient agar
PFU	Plaque forming unit
PBS	Phosphate-buffered saline
MOI	Multiplicity of infection
CFU	Colony forming unit
ICTV	International Committee on Taxonomy of Viruses
GBDP	Genome-BLAST Distance Phylogeny
CDS	Coding sequences
AMP	Ampicillin
GEN	Gentamicin
COL	Colistin
FICI	Fractional inhibitory concentration index
