# Supplementary material for: Isolation of Lytic Bacteriophages of Escherichia coli and Their Combined Use with Antibiotics Against the Causative Agents of Colibacillosis in Calves
Source: Vet Sci. 2025 Aug 26;12(9):817. doi: 10.3390/vetsci12090817 (PMC12474050; doi:10.3390/vetsci12090817)
Supplement: Supplementary file 1 [file vetsci-12-00817-s001.zip › vetsci-3831644-supplementary/Table S1.pdf]

Table S1. Antibiotic susceptibility of *E. coli* strains.

№	ID E. coli	Ampicillin (AMP-10 µg)	Tetracycline (TET-30 µg)	Gentamicin (GEN-10 µg)	Trimethoprim/ Sulfamethoxazole (SXT-25 µg)	Enrofloxacin (ENR-5 µg)	Colistin (COL-10 µg)	Amoxicillin/ Clavulanate (AMC-30 µg)	Florfenicol (FFC-30 µg)
1	1MC	S*	S	S	S	S	S	S	S
2	4MY	S	S	S	S	S	S	S	S
3	8MY	I	S	S	S	S	S	S	S
4	9MY	S	S	S	S	S	S	S	S
5	12MC	S	S	S	S	S	S	S	S
6	16-1 ML	S	S	S	S	S	S	S	S
7	16-1 P	S	S	S	S	S	S	S	S
8	16-2 CF	R	R	R	R	R	S	I	S
9	17-1 YF	S	R	S	R	R	S	S	S
10	18 YML	R	R	S	R	S	S	I	R
11	19-AY	R	R	S	S	S	S	R	R
12	19-BYF	R	R	S	S	S	S	I	R
13	20-1YF	R	R	S	R	S	S	I	R
14	20-2Y	R	R	S	R	S	S	I	R
15	21-1C	S	S	S	S	S	S	S	S
16	21-2YF	S	S	S	S	S	S	S	S
17	22-1YF	R	I	S	S	S	S	S	R
18	23-1YF	R	R	S	R	S	S	R	S
19	23-2Y	R	S	S	S	S	S	S	S
20	24CF	R	R	R	R	R	S	I	S
21	25-1CF	R	R	R	R	R	S	I	S
22	25-2P	R	S	R	S	S	S	S	S
23	25-2W	S	S	S	S	S	S	S	S
24	26	R	R	R	R	R	S	I	R
25	27	R	R	R	R	R	S	I	R
26	32	R	R	R	R	R	S	S	R
27	33	R	R	R	R	R	S	R	R
28	35	R	R	R	R	R	R	R	R
29	37	R	R	R	S	S	S	I	R
30	38	R	R	R	R	R	S	I	R
31	39	S	S	S	S	S	S	S	S
32	44	S	S	S	S	S	S	S	S
33	45	S	S	S	S	S	S	S	S
34	46	S	S	S	S	S	S	S	S
35	48	S	S	S	S	S	S	S	S
**Number of resistant strains**		**20**	**18**	**11**	**14**	**10**	**1**	**15**	**13**

*R – resistance; I – intermediate resistance; S – susceptibility. *Escherichia coli* strains were isolated from samples obtained from the upper layers of fresh feces of calves with clinical signs of diarrhea, kept on livestock farms in the Almaty region, Republic of Kazakhstan.
