# Supplementary material for: Isolation of Lytic Bacteriophages of Escherichia coli and Their Combined Use with Antibiotics Against the Causative Agents of Colibacillosis in Calves
Source: Vet Sci. 2025 Aug 26;12(9):817. doi: 10.3390/vetsci12090817 (PMC12474050; doi:10.3390/vetsci12090817)
Supplement: Supplementary file 1 [file vetsci-12-00817-s001.zip › vetsci-3831644-supplementary/Table S2.pdf]

Table S2. Interpretation breakpoints of the various antimicrobial agents used in the study

| Antibiotic | Concentration, (µg) | Breaking Point (mm) | |
|---|---|---|---|
| | | Sensitive (S) | Resistant (R) |
| Ampicillin (AMP)* | 10 | ≥ 17 | ≤ 13 |
| Tetraciclin (TET)** | 30 | ≥ 19 | < 17 |
| Gentamicin (GEN)** | 10 | ≥ 18 | < 16 |
| Trimethoprim/ Sulfamethoxazole (SXT)** | 25 | ≥ 16 | < 10 |
| Enrofloxacin (ENR)** | 5 | ≥ 19 | < 19 |
| Amoxicillin/ Clavulanic acid (AMC)** | 30 | ≥ 21 | < 14 |
| Florfenicol (FFC)** | 30 | ≥ 19 | < 19 |
| Colistin (COL) | 10 | The susceptibility of the bacterial isolate to colistin was determined by the broth disk elution method according to CLSI recommendations. | |

* Breaking point indicated according to CLSI (Clinical and Laboratory Standards Institute), 2023.
** Breaking point indicated according to CASFM (Comité de l'antibiogramme de la Société Française de Microbiologie, Recommanda-tions Vétérinaires), 2021.
